# Patient Perspectives on the Development of a Novel Mobile Health (mHealth) Application for Dietary Supplement Tracking and Reconciliation—A Qualitative Focus Group Study

**DOI:** 10.1177/21649561221075268

**Published:** 2022-01-27

**Authors:** Elana Post, Keturah Faurot, Zachary O. Kadro, Jacob Hill, Catharine Nguyen, Gary N. Asher, Susan Gaylord, Amanda Corbett

**Affiliations:** 1 Eshelman School of Pharmacy, 2331 The University of North Carolina at Chapel Hill, Chapel Hill, NC, USA; 2 School of Medicine, Department of Physical Medicine and Rehabilitation, Program on Integrative Medicine, 6797 The University of North Carolina at Chapel Hill, Chapel Hill, NC, USA; 3 School of Medicine, Department of Family Medicine, 6797 The University of North Carolina at Chapel Hill, Chapel Hill, NC, USA

**Keywords:** qualitative research, integrative medicine, dietary supplements, mobile health, medication reconciliation

## Abstract

**Background:**

More than 170 million adults use dietary supplements (DS) in the United States, which can have both benefit and harm to patient health. DS use is often poorly documented in the medical record and can pose health risks if not properly communicated with providers. Reasons for poor DS documentation include low disclosure rates, time constraints of clinical encounters, and providers’ failure to inquire about DS use. This study was conducted to assess patients’ views on the facilitators and barriers to using a mobile health (mHealth) application (app) to collect and share DS information with their healthcare providers.

**Methods:**

Utilizing a theory-based conceptual model, we conducted 7 patient focus groups (FGs) to assess opinions on DS safety, provider communication, comfort with technology use, and our proposed mHealth app. Participants were recruited from the general public and through patient advisory groups. Patient views will inform the creation of an mHealth app to improve DS patient-provider communication and tracking and reconciliation in the electronic medical record (EMR).

**Results:**

Overall, participants believe their DS information is inaccurately represented in the EMR, leading to safety concerns and negatively impacting overall quality of care. Participants desired an app designed with (1) Health Insurance Portability and Accountability Act (HIPAA) compliance; (2) ease of use for a variety of technical efficacy levels; (3) access to reliable DS information, including a DS-drug interaction checker; and (4) integration with the EMR.

**Conclusion:**

An app to simplify and improve DS entry and reconciliation was of interest to patients, as long as it maintained health autonomy and privacy and possessed key valuable features.

## Introduction

In the United States, dietary supplements (DS) are widely used, especially among patients with acute and chronic illnesses.^1-3^ DS use is highly prevalent among cancer survivors (70.4%),^4^ ranging from 70% in breast cancer^5^ to 85% in gynecologic cancer,^6^ as well as among patients with multiple chronic conditions (50%)^7^ including hypercholesterolemia (30%), hypertension (28%), and diabetes (25%).^8^ Furthermore, concomitant use of DS with prescription medications is common, ranging from 34% in all age groups^9^ to 66% among older adults.^10^ In patients with chronic disease, who often take multiple prescription medications, some supplements are unsafe due to supplement-drug interactions (SDI).^11-13^

DS are often presumed by patients to have a low potential for harm, contributing to low rates of disclosure in medical encounters.^14^ DS manufacturers are not required to submit safety and efficacy data to the Food and Drug Administration (FDA) prior to marketing, and quality control can vary widely among manufacturers; therefore, patients’ perceptions of safety may be misplaced. Problematic DS products are recalled via voluntary post-marketing reporting, but the FDA estimates they are notified of < 1% of adverse effects associated with DS use.^15-17^ Additionally, DS can have important interactions with prescription medications.^18^ Despite recommendations by both the Joint Commission and the American Society of Health-System Pharmacists, many health systems lack policies regarding DS use and monitoring.^19^

Documentation of DS use may be poor for several reasons including (1) low rates of disclosure by patients,^20,21^ (2) time-sensitive nature of clinical encounters,^22^ and (3) providers not asking or supporting DS use.^21^ For example, a study of hospitalized patients at a tertiary center found only 6% of DS users were asked, had disclosed, and had documentation of DS use in the electronic medical record (EMR) during their inpatient stay.^20^ Another study found 49% of DS users discussed at least 1 supplement with their provider but disclosed only 34% of total DS products used.^21^ Two studies of hospitalized patients found that physicians inquired about DS use roughly 20% of the time.^18,20^ Reasons for lack of inquiry include short clinical encounters and the multidisciplinary approach to reconciliation, creating confusion on role responsibility.^20-22^ Poor DS documentation and patient-provider communication impedes identification of important medication safety issues such as SDI, DS-disease interactions, adverse events, and overdosing.^20,21,23^ New methods are needed to simplify and improve the process of DS tracking and reconciliation.

A mobile health technology (mHealth) application (app) could facilitate entry of medical information into the EMR.^24^ For example, bar-code scanning of DS products could reduce the time and errors associated with manual data entry. Additionally, a current and accurate list of DS products may encourage patient-provider communication about supplements.^24^ The app would be useful for organizing key DS information, including brand name, ingredients, and recommended dose, to communicate with providers.

This qualitative study aimed to interview representative patients via focus groups (FGs), collect and analyze their responses, and describe patients’ perceptions on the safety and risk associated with DS use and their experience of DS tracking and reconciliation, as well as their views on using an mHealth app to collect and share DS information with their healthcare providers. Patient input will inform the creation of an mHealth app that will collect DS information in a way that is congruent with patient’s needs and desires, and that will facilitate improved communication about DS use with their healthcare providers. The study takes into consideration existing mHealth apps along with their strengths and deficiencies.

## Methods

### Study Design and Conceptual Model

**Figure 1.**
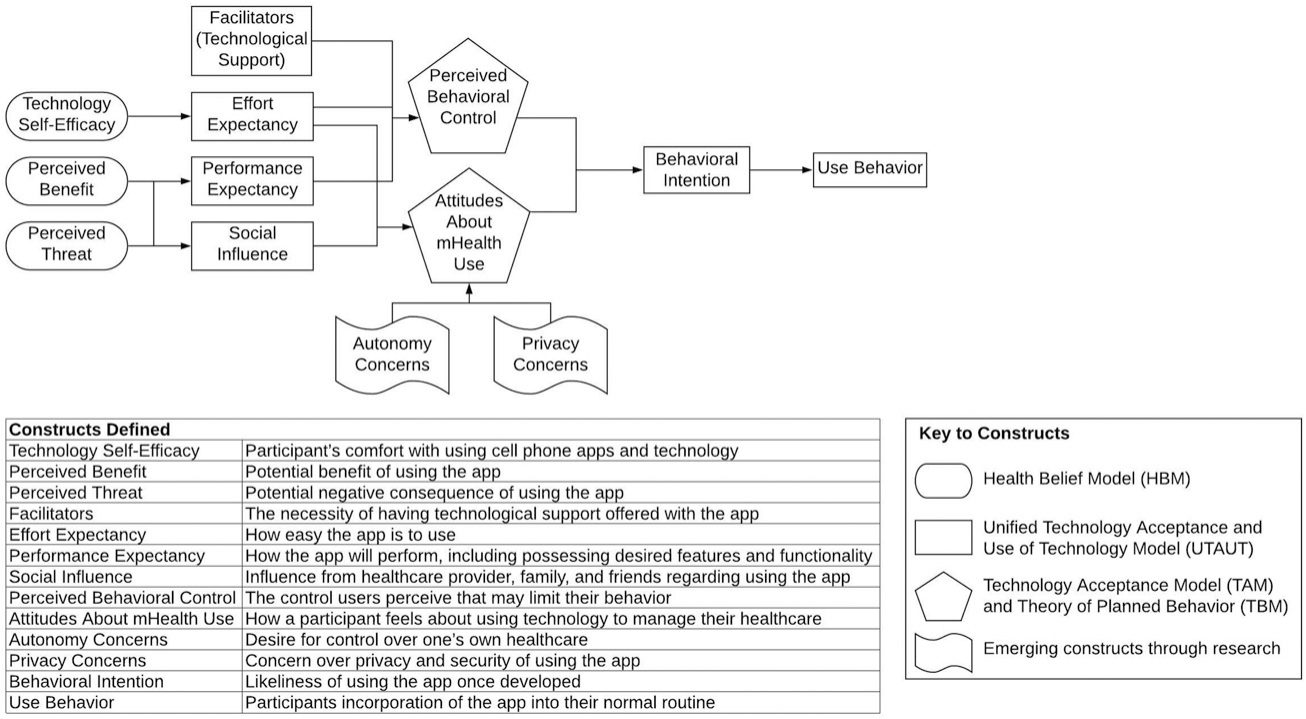
Conceptual model for study of attitudes and intention to use dietary supplement mHealth app. Model created on Lucid.app. Ovals describe constructs from the Health Belief Model (HBM): Technology Self-Efficacy, Perceived Benefit, and Perceived Threat.^28^ Rectangles represent original concepts from the Unified Technology Acceptance and Use of Technology (UTAUT) model: Facilitators (Technological Support), Effort Expectancy, Performance Expectancy, Social Influence, Behavioral Intention, and Use Behavior.^27,30^ Pentagons represent constructs from the Technology Acceptance Model (TAM) and Theory of Planned Behavior Model (TBM): Perceived Behavioral Control and Attitudes About mHealth Use.^30^ Banners are important constructs that have emerged through investigations from other researchers: Autonomy Concerns and Privacy Concerns.^27^ All the constructs lead to behavioral intention and use behavior which is the main goal of our mHealth app development.

We conducted a qualitative FG study using an applied social anthropology approach to create a conceptual model (Figure 1).^25^ FGs were conducted in person or via a secure teleconference interface. Data collection continued until we reached data saturation, which was defined when we heard no new information with subsequent FGs. This study was approved by the Institutional Review Board.

### Conceptual Model

Our conceptual model (Figure 1) identified key constructs that aimed to predict a person’s intention to use our DS mHealth app. Our model was based on the Unified Technology Acceptance and Use of Technology (UTAUT) model, a popular model for evaluating consumer technology usage behavior that is based on 4 key constructs: performance expectancy, effort expectancy, social influence, and facilitating factors.^26,27^ We enriched the model with health behavior theories, including the Health Belief Model (HBM) (Perceived Threat and Benefits), the Theory of Planned Behavior (TPB) (Social), the Technology Acceptance Model (TAM), and key factors that emerged in the literature as important (Autonomy and Privacy).^28-30^

Both commonly employed technology acceptance models, the TAM^31-33^ or its extension^34^ and the UTAUT,^26,35^ are derived from and often recombined with the Fishbein and Ajzen’s TPB. TPB posits that behavioral intention drives the adoption of behaviors, and is itself influenced by a person’s attitude toward the behavior, the degree to which a person feels that important people approve or disapprove of the behavior, and the degree to which a person feels that they have control over the behavior.^36^ We added factors derived from other health behavior models, notably self-efficacy, and perceived benefit, and perceived threat from the HBM,^37^ as well as attitude and perceived behavioral control from the TPB to capture potentially important drivers of DS mHealth app use. We defined self-efficacy as the perceived ability to interact with technology in general. Perceived threat (susceptibility and severity) indicates the degree to which potential users believe there are risks associated with supplement use, such as drug-supplement interactions. Perceived benefit indicates the extent to which potential users believe that communicating dietary supplement use to a clinician will reduce those risks. Attitudes measure the overall perceived value of the mHealth app to potential users. Perceived behavioral control measures the degree to which potential users believe that they could use the described mHealth app (see Supplementary Table S1 in Online Resources to view the FG guide linking the model constructs to the questions). The conceptual model and defined constructs can be found in Figure 1. Our model led us to the following research questions:

(1). What are patients’ experiences with sharing DS information with their healthcare providers?
(2). What are patients’ views on the safety of DS?
(3). What factors do patients see as important in determining whether they would use an mHealth app to record and share DS use with their healthcare providers?
(4). What do patients perceive as the facilitators and barriers to use of a DS app?

### Population and Recruitment

We used a purposive sampling technique, aiming for individuals who are representative of our patient population. Patient advisory group (PAG) members from multiple departments of an academic medical center were recruited into 4 FGs. To improve participant diversity, additional FGs were conducted with members of the general public within 50 miles of Chapel Hill and recruited through ResearchMatch.org, an organization that enables people to volunteer for studies of interest. Potential participants received an email describing the goal of the study. All adults who responded to the email were eligible to participate regardless of their health status or use of DS. FGs were limited to < 7 participants per group to enable constructive conversation. Compensation was $25 for participants in the FG.

### Data Collection

FGs were led by experienced facilitators (KF and SG) and supported by additional study staff. Data were collected via audio recording and supplemental notes with information housed in a secure tracking database. Prior to the start of each FG, a consent form was read aloud, and participants were provided with an opportunity to ask questions, confirm verbal understanding, and provide verbal consent. Ground rules for respectful discussion were established, and participants were cautioned to maintain confidentiality.

The FG discussion guide included 13 questions with follow-up prompts (see Supplementary Online Resource). Questions included queries about the perception of DS safety and risks, communication with providers, and comfort with use of technology. After these initial discussions, a presentation of the possible app design was shared. Participants were invited to comment on app features, including (1) linking to a database dedicated to DS information, (2) bar-code scanning, (3) capturing dose and frequency, and (4) linking to the EMR.

To protect privacy, participant names and identifiable information were not recorded. Participants provided demographic information including age, gender, race/ethnicity, education, and personal DS use.

### Data Analysis

FGs were audio recorded and transcribed verbatim. Analysis used a postpositivist interpretive strategy. Qualitative analysis was conducted using ATLAS.ti (*Version 8, Scientific Software Development GmbH, Berlin, Germany*). Team members (EP, ZK, KF, and CN) generated code groups based on model constructs (e.g., “performance expectancy” and “effort expectancy”). Individual codes within code groups were driven by data from the FG discussions. Two investigators (EP and ZK) independently coded each transcript and created the individual codes. The investigators used an iterative code comparison process to reduce interpretation bias. During the final analysis, the entire team compared codes across all transcripts, finalized the code book, and measured frequency of codes mentioned by participants as well as the emphasis given to each by participants in context of the conversation (Supplementary Table S2 in Online Resources).

### Researcher Characteristics

AC, GA, KF, SG, and JH are experienced integrative health researchers. KF, SG, and JH are experienced in qualitative research. No researchers had prior relationships with FG participants.

## Results

### Study Participants

From August 2019 to June 2020, 6 FGs (and 1 interview) were held with a total of 24 participants (Tables 1 and 2). After 6 FGs, we reached data saturation. Of recruited participants, 4 (2 PAG members and 2 members of the general public) who expressed interest in the FG failed to attend. One person was confused about the time and missed the meeting. In most cases, however, reasons for non-attendance were not given, potentially introducing bias. Of the participants who completed the FGs, 67% were female, 57% non-Hispanic White, and 63% were PAG members. All reported post-secondary education and 88% reported DS use. We did not ask participants to disclose their health information, but we do know which PAG they came from (see Table 1), and some members disclosed health information during FG discussions.

**Table 1.** Baseline Demographics and Characteristics of Focus Group Participants.

Characteristic	FG participants (N = 24)
Median age (range)—yr	57 (28-77)
Gender—no. (%)	
Male	8 (33)
Female	16 (67)
Race and ethnicity—no. (%)	
Non-Hispanic White	14 (58)
Non-Hispanic Black, African, or African American	6 (25)
Hispanic or Latino	3 (13)
Asian	1 (4)
Highest level of education—no. (%)	
Grade school, high school, or GED	0 (0)
Associate or technical degree	1 (4)
Bachelor’s degree	7 (29)
Master’s degree	9 (38)
Doctoral degree	7 (29)
Dietary supplement use—no. (%)	
Yes	21 (88)
No	2 (8)
N/A	1 (4)
Patient population—no. (%)	
UNC Physical Medicine and Rehabilitation PAG	3 (13)
UNC Family Medicine PAG	5 (21)
UNC Lineberger Comprehensive Cancer Center PAG	7 (29)
General public	9 (38)

**Table 2.** FG Session Characteristics.

FG #	# of participants	FG length, minutes	Location
1	6	37	In-person in a clinic conference room
2	6	97	In-person in a clinic conference room
3	3	59	In-person in a clinic conference room
4	3	87	In-person in a clinic conference room and virtual (split)
5	3	62	Virtual
6	1	35	Virtual
7	2	45	Virtual

### Focus Group Themes

In the analysis, the following themes were related to or emerged from our model: (1) concern about DS safety and inaccurate documentation, (2) advantages of mHealth use, (3) attitudes to mHealth use, and (4) behavioral intention and use behavior.

### Perceived Threat: Concerns About DS Safety

Some participants expressed concern about DS quality and safety, including wariness of DS advertisements (e.g., false or misleading information in advertisements). They identified the potential for SDI and highlighted the importance of sharing DS use with their providers: “[Supplements] effect the way [drugs] work in your body… It should be particularly concerning if you are not sharing that with someone on your team… It needs to get into your medication record.”

Many participants stated concern that their DS were not properly recorded in the EMR due to difficulty entering DS into the system, insufficient time during visits, or the perceived lack of importance of these products by providers. Participants were not always asked about their DS use, and even when asked, they were not queried about details:

“Whenever I go to the physician’s office, they always ask me if I’m taking the medications that are on the list, but never ask me if I’m taking any supplements.”

“[I don’t] think anything got in there about how long you are taking or how often.”

They also discussed doctors’ lack of training regarding DS:

“I’m very concerned that there’s nothing in doctors’ normal training that teaches them to understand interactions or benefits or anything having to do with supplements.”

“If [a provider] doesn’t know [DS information] I don’t think she will have the time to look it up to see if there’s any interaction”

### Perceived Benefits: Advantages to mHealth App Use

Perceived advantages of mHealth app use emerged as well. Participants felt an app would enhance patient-provider communication by enabling disclosure of DS use to providers, as well as free up time in the clinical encounter for meaningful discussion around DS use. They believed the app could overcome documentation challenges by ensuring accurate input of the DS brand, dose, and frequency, and would empower the patient in taking control of their health. Having access to the list on their phone, with appropriate reminders, would ensure the list is always kept up-to-date and accurate. Participants shared:

“It will make it easy for both the patient and the healthcare provider in that the records would be in a central place that either one of us can get to.”

“I think that it helps the patient provide more information to [the provider] in a more precise way and they can get to information, exact dosage, and name of the [product].”

Another perceived advantage of the app was its capability of providing reliable DS information for both the patient and provider. Participants felt that the app should include readily accessible and reliable DS information including existing clinical data, recommended doses, and known interactions: “Ideally internal to that app is all of the information about the interaction effects of drugs and supplements.”

Having readily available and reliable DS information regarding products patients are taking would inform patient and provider conversations: “I think it would educate me and inform my conversation with my doctor to ask if I should take XYZ at the same time.”

Some participants recognized the lack of training about DS in conventional medicine and hoped the app would promote team-based care by enabling communication with providers specialized in DS (e.g., pharmacists, dietitians, and naturopathic doctors). Integration into the EMR would allow identification of safety concerns, such as inappropriate doses and SDI, and result in improved care:

“It’ll be easier for [providers] to say, ‘Okay, I see you're taking vitamin D, this milligram is based on whatever labs we've done, [and I] want you to increase or decrease,’ it just would make it easier for that provider to provide care in general.”

### Attitudes About mHealth App Use

#### Autonomy Concerns

Some participants were wary of sharing some DS, such as the use of cannabidiol products, products that replace positive health behaviors (e.g., weight-loss supplements to replace a healthy lifestyle), or products for memory enhancement. Participants acknowledged they would be hesitant to discuss DS with providers if they expected disapproval. When asked about sharing DS information with a provider, one participant stated:

“It’s not easy because sometimes they don’t agree with you. I have provided some to my primary care provider, however, I did not provide all of them, because there is a hesitation always that they don’t believe in this and they don’t believe in that”

However, some participants were interested in an app that could improve their ability to consistently take their DS and monitor their response: “I would like to use something like this to keep track of what I'm taking and how often I'm taking.” Others hoped for maximum flexibility regarding reminders and push notifications.

#### Privacy Concerns

Concerns related to mHealth app use stemmed from overall attitudes about technology. Participants expected that the app would include standard HIPAA-compliant security features: “If it wasn’t covered under HIPAA, I probably wouldn’t use it.” Participants also were wary about receiving unsolicitated advertising as a consequence of a leaked list.

With the assumption of HIPAA-compliant security features, most participants were not worried about the privacy of their information, particularly if no other personal health information was in the app:

“I personally would be more concerned with people seeing my prescribed drug list than I would with people seeing my dietary supplement drug list.”

#### Technology Access and Self-Efficacy

Access to the technology needed for the app by older or lower socioeconomic status (SES) users was a concern:

“It's not available then to people who are poor and live in rural areas or people who live in public housing areas, they just don't have the money to buy that kind of phone, so cost is a barrier.”

Additionally, they were concerned about the technology self-efficacy for people who would not be able to navigate the app on their own: “I’m not so sure it would be easy for an elderly or aging person who is not already electronically savvy to understand and to be consistent with [using the app].” For these users, point-of-care bar-code scanning in the provider’s office was suggested.

#### Facilitators (Technological Support)

A few felt technical facilitators or tech support would be necessary to help users navigate the app, while most felt it was not necessary: “It should be easy to begin with, [users] shouldn’t have to go to tech support.”

### Behavioral Intention and Use Behavior: Factors to Influence mHealth App Use

#### Effort Expectancy and Performance Expectancy

Participants who regularly took DS stated they would be likely to use the app, whereas those who did not take DS regularly were less inclined. Participants expressed higher likelihood of utilizing the app if it possessed key features and functionality to meet their individual needs. Almost all the participants desired the app to connect with the EMR, to be accessible to both themselves and providers: “I would be likely to use it if I knew it would tie into the existing system that my doctor uses.”

#### Social Influence

When asked about the impact of support for the app from their doctor, participants stated they would use the app regardless of support from their provider if they perceived benefit from the app: “I would use it either way and wouldn’t try to push it on the doctor but would hope he shows some sort of interest.”

## Discussion

In general, participant responses within the FGs were consistent with model-based expectations—we found that responses leading to codes easily fit into the code groups defined by the constructs. The most common constructs, as determined through frequency in coding and language utilized by participants, were effort expectancy, self-efficacy, performance expectancy, and perceived benefit. Participants desired an app that was easy to use with a user-friendly interface. They were more likely to report they would use the app if their baseline technology self-efficacy was high (i.e., they had confidence in their ability to navigate the technology) and if they perceived greater personal potential benefit from using the app (i.e., DS users were more likely to use it than non-users).

Participants desired an app to contain features such as a drug-DS interaction checker, access to reliable DS information, and connection to the EMR, while maintaining their health autonomy (i.e., allowing them to decide on what they share with the provider). They desired the ability to tailor the app experience to their personal needs (e.g., keep track of their DS list and send reminders to take DS).

Constructs that did not hold as much weight in participants’ intention to utilize the app included privacy and social influence. Participants acknowledged the high prevalence of health information housed electronically and expressed no concern using the app if it contained standard HIPAA-compliant privacy measures. Although participants preferred support from their provider for using the app, they stated that they would continue to use it if they perceived health benefits.

Although, to our knowledge, this is the first study to examine the potential willingness of participants to use a DS mHealth product, other studies have evaluated acceptance of mHealth technology. In a study testing a mock-up of a COPD mHealth app, similar to our study, participants valued flexibility in the use of the product with additional feature choices.^38^ Also, similarly, participants felt that the app would enhance communication with their providers.^38^ Most of the other studies, also using qualitative methods, focused on the needs of older adults in the use of a variety of product types including medication adherence apps,^39^ mental health support apps,^40^ and general technology acceptance.^41^ In aggregate, participants in these studies indicated a preference for apps that were simple to use, easy to learn, and secure.

We believe our proposed app is different than others on the market. Through an extensive market analysis in partnership with UNC-Chapel Hill’s Office of Technology Commercialization, our team identified 5 mHealth apps that competed with our proposed app in our 4 key features (bar-code scanning, comprehensive DS database, DS list generation, or EMR interface): Amlia, Medisafe, MangoHealth, CareZone, and Dosecast. However, of these 5, none of them contained all of our proposed features. Therefore, our app fills the need of providing all of these desired features and functionalities to users in one streamlined app.

### Limitations

The primary study limitation was the study sample (sample size, diversity, and age). We were limited in our capacity to recruit and speak to a fully diverse population, since we were limited geographically to central North Carolina. Most of our study sample included PAG members, which are “expert patients” that are self-selected to support the healthcare system, and ResearchMatch volunteers, which attracts general public members who are interested in research and may not represent typical users of the app. We did not identify participants’ health statuses and the ways in which their health status may have influenced their responses or decision to participate in the study is unknown. Our study population predominantly included a higher level of education, and we did not speak with many less educated or elderly participants. Since DS use is higher among those of higher SES status,^42^ and higher SES is likely correlated with higher education, it can be inferred that our study results are not fully representative. Additionally, persons with the latter demographic characteristics are at higher risk for having low technology access and self-efficacy and potentially complex medication regimens, and their opinions should be captured. We did not have a significantly diverse racial participant population in our study. As it has been suggested that Asian Americans can possibly have higher DS use than other racial/ethnic groups,^43^ it is important to speak to as diverse racial groups as possible. It is possible we would have identified additional themes with a more varied population (i.e., our belief that we had reached saturation may have been false since a more diverse population could have introduced varied ideas not mentioned by our current population), and our results may not be generalizable to these populations. However, PAG members ensured advocacy for these participants and their potential views, and we attempted to address this deficiency in our sampling by inviting participation by diverse members of the general public. PAG members specifically brought our attention to the needs of those with lower technology self-efficacy. Additionally, our study’s sample size had twice as many women than men. Since women tend to have higher DS use than men,^44^ it can be inferred that our results are skewed more positively and supportive of our proposed mHealth app. However, including more women can potentially capture more of our intended mHealth app users.

An additional limitation of FG studies can include participants’ hesitation to discuss sensitive topics, such as extent or type of DS use (e.g., cannabis products and memory enhancement). Participants may also be influenced by other participants’ responses. However, since our participants were representing patients other than themselves, they were not hesitant to bring up and discuss sensitive issues. Potential disclosure hesitation by participants was considered during our analysis.

Although we attempted to conduct our FGs with relatively equal participation, FG #6 and #7 were conducted virtually with just 1 and 2 people, respectively. To remedy this low turn-out, the moderator included talking points from previous FG participants to stimulate a conversation that may have occurred with more participants. Despite low numbers, the FGs lasted a comparable amount of time to the other FGs, indicating thorough and thoughtful discussion with the moderator and new ideas were presented by these individuals. Reasons for the low recruitment number can be attributed in part to COVID-19.

Another study limitation was conducting the last FGs via teleconferencing due to COVID-19. Having online FGs, rather than in person, has the potential to limit the natural flow of conversation. However, the FGs conducted over Zoom did not seem to limit conversation amongst participants compared to our other FGs.

A final limitation of implementing our proposed mHealth app into the health system is potentially needing physician or administrative approval and reducing DS stigma to encourage patients and providers to use the app together. This would require long-term systemic change, such as increasing physician DS training and team-based care. This limitation is further explored in our congruent study interviewing healthcare providers.

## Conclusions

An app to simplify and improve DS entry and reconciliation was of interest to patients if it maintained health autonomy and privacy and possessed key valuable features. We found that patients were more likely to be interested in using an mHealth app containing the following elements: (1) HIPAA-compliance; (2) ease of use for a variety of technical self-efficacy levels; (3) access to reliable DS information, including a DS-drug interaction checker; and (4) integration within the EMR. Current apps do not contain all 4 elements that we heard were important from end users. We believe that if the app meets the above criteria, patients will use it to track and communicate their DS use with their providers for enhanced DS reconciliation.

Because patients may be uncomfortable with their own use of certain DS products (e.g., for weight loss or memory), and may perceive physicians are not trained in DS, future research is needed to address the impact of these factors regarding implementation of the DS app. Increasing access to clinicians who are trained in DS (e.g., pharmacists, dieticians, and naturopathic doctors) may be important to implementing the app. The current study explored patient attitudes, experiences, and beliefs around DS tracking and reconciliation and their likelihood of adopting an mHealth app to track and communicate DS use with their providers. Future implementation of a DS mHealth app would further require support, adoption, and integration from providers. To that end, our study team also interviewed providers to understand their attitudes, experiences, and beliefs about DS tracking and reconciliation, and our proposed app: findings from our companion study will serve as a complement to the current work, with the larger goal of integrating both patient and provider perspectives in the investigation of this important gap in patient care.

## Supplemental Material

sj-pdf-1-gam-10.1177_21649561211044693 – Supplemental Material for Patient Perspectives on the Development of a Novel Mobile Health (mHealth) Application for Dietary Supplement Tracking and Reconciliation—A Qualitative Focus Group Study Click here for additional data file. Supplemental Material, sj-pdf-1-gam-10.1177_21649561221075268 for Patient Perspectives on the Development of a Novel Mobile Health (mHealth) Application for Dietary Supplement Tracking and Reconciliation—A Qualitative Focus Group Study by Elana Post, Keturah Faurot, Zachary Kadro, Jacob Hill, Catharine Nguyen, Gary N. Asher, Susan Gaylord, Amanda Corbett in Global Advances in Health and Medicine
