# Supplementary material for: Patient Perspectives on the Development of a Novel Mobile Health (mHealth) Application for Dietary Supplement Tracking and Reconciliation—A Qualitative Focus Group Study
Source: Glob Adv Health Med. 2022 Jan 27;11:21649561221075268. doi: 10.1177/21649561221075268 (PMC8862130; doi:10.1177/21649561221075268)
Supplement: sj-pdf-1-gam-10.1177_21649561211044693 – Supplemental Material for Patient Perspectives on the Development of a Novel Mobile Health (mHealth) Application for Dietary Supplement Tracking and Reconciliation—A Qualitative Focus Group Study [file sj-pdf-1-gam-10.1177_21649561221075268.pdf]

Patient Perspectives on the Development of a Novel Mobile Health (mHealth) Application for Dietary Supplement Tracking and Reconciliation – A Qualitative Focus Group Study

Global Advances in Health and Medicine, Supplemental Information

Elana Post, PharmD[1]; Keturah Faurot, PhD, PA, MPH[2]; Zachary Kadro, ND[2]; Jacob Hill, ND, MS, FABNO[2]; Catharine Nguyen, PharmD[1]; Gary N. Asher, MD, MPH[3]; Susan Gaylord, PhD[2]; Amanda Corbett, PharmD[1]

[1]*Division of Pharmacotherapy and Experimental Therapeutics, University of North Carolina Eshelman School of Pharmacy, Chapel Hill, NC, USA*

[2]*Department of Physical Medicine & Rehabilitation, Program on Integrative Medicine, University of North Carolina at Chapel Hill, Chapel Hill, NC, USA*

[3]*Department of Family Medicine, University of North Carolina at Chapel Hill, Chapel Hill, NC, USA*

Corresponding Author:
Amanda Corbett
ahcorbet@email.unc.edu
The University of North Carolina, Eshelman School of Pharmacy
CB# 7569, 324 Beard Hall
Chapel Hill, NC 27599-7355

Focus Group Introduction Script

Thank you for taking the time to join this discussion today. My name is Kim and I will be the moderator for this focus group. The purpose of this discussion is to gather your opinions regarding tracking and reconciliation of dietary supplements. The definition of a dietary supplement is, "*A product that is intended to supplement the diet. A dietary supplement contains one or more dietary ingredients (including vitamins, minerals, herbs or other botanicals, amino acids, and other substances) or their components; is intended to be taken by mouth as a pill, capsule, tablet, or liquid; and is identified on the front label of the product as being a dietary supplement.*" We will discuss the medication reconciliation process during patient visits, and how dietary supplements are, or are not, included in that process. For our study, we define medication reconciliation as the process of comparing a patient's medication list in their medical chart to the medications that the patient has actually been taking. This reconciliation is done to avoid medication errors such as omissions, duplications, dosing errors, or drug interactions.

You will be provided with a $25 gift card for your participation today, and food will be provided.

This focus group is being conducted to support a larger study on improving the documentation of dietary supplements in a patient's chart. Both the focus group and the subsequent research study are being conducted by a team of researchers here at the University of North Carolina, Chapel Hill.

Before we start, I would like to remind you that there are no right or wrong answers in this discussion. I am interested in knowing what each of you think. Please be honest and share what you think, even if you don't agree with others in the group. It is very important that I hear all of your opinions, but you may choose not to answer a question at any time.

Let's start by going around the circle and have each person introduce themselves. I would like each of you to state either a first name or a nickname that you would like to use for the discussion. Your name will not be used when we summarize group responses, or in any written reports.

Let's make a list of rules to guide our discussion today. I would like for us, as a group, to agree that what is shared here today stays within our group. In other words, to make sure that everyone feels

comfortable sharing their opinions, I want us to agree that we will not talk about what we discuss today with people who were not in this group. We have a few other ground rules to cover, and let me know if you have any others that you would like to add.

Ground Rules

- Everything we talk about today is private
- Use first names or nicknames only when referring to other participants
- There are no right or wrong answers
- It is important that we respect everyone’s opinions, even if they are different from our own
- We want to hear from everyone

Can we as a group agree on these ground rules? Would you like to add any other ground rules? Do you have any questions before we begin recording the discussion? Does everyone agree to having the recorder turned on?

Focus Group Discussion Guide

1. Do you think it is common for people to use dietary supplements, such as vitamins, minerals, herbal supplements, or food-based supplements?
    a. *Follow-up:* Which type of supplements do you think are most commonly used?
2. Do you think supplements can affect the way medications work in the body?
    a. *Follow-up*: How concerned are you about the risks associated with dietary supplements, particularly those your healthcare provider does not know you are taking?
3. Do you think it is important for a person's care team, including their physician, to know if they are using supplements?
    a. *Follow-up:* How do you feel about sharing information about your dietary supplement use with your provider?
    b. *Follow-up:* Do you think it is a hard thing for people to discuss with their provider?
    c. *Follow-up:* Are there health habits you would not want to share?
4. How well do you think healthcare providers document supplement information in a patient's medical chart (reconciliation)?
    a. *Follow-up:* If not well, why might the information be inaccurate?
    b. *Follow-up:* Do you think it is hard for healthcare providers to keep track of patient's supplement use? Why or why not?

Now we will ask questions about our proposed mobile application. We anticipate creating an app that people can use at home to document their dietary supplements. The app would enable them to scan the supplement barcode and create a list that could be shared with their provider or uploaded into the electronic health record. (Show diagram)

5. How comfortable are you with cell phone apps in general?
6. Do you think it would be helpful to have mobile app that scans a supplement's barcode?
    a. *Follow-up:* As described, how easy do you think the app might be to use?

    b. *Follow-up:* What might it easier to use?
    c. *Follow-up:* What barriers do you see to using the app?

7. How important do you think it would be to have tech support to help you figure out how to use the app?
    a. *Follow-up:* How important would tech support be to troubleshoot with you if there are problems?
8. What do you think the dietary supplement mHealth app could do for you?
    a. *Follow-up:* How can the process of documenting supplement use in a patient's chart be easier for patients and healthcare providers?
9. How do you think your healthcare provider will feel about you use of dietary supplement app to share your supplement list?
10. How concerned would you be about privacy in using the app?
    a. *Follow-up:* How would you feel if your dietary supplement information was shared without your knowledge?
11. If the dietary supplement app were available today, how likely would you be to use it?
    a. *Follow-up:* Why or why not?
12. How do you feel about the proposed dietary supplement app?
    a. *Follow-up:* What would an ideal mobile app for users of dietary supplements look like to you?
13. Is there anything else you would like to add regarding collection of medication and dietary supplement information?

Table S1 Constructs Being Measured by FG Discussion Questions

Self-efficacy	How comfortable are you with cell phone apps in general?
Effort expectancy	As described, how easy do you think the app might be to use? What might make it easier to use?
Facilitators	How important do you think it would be to have tech support to help you figure out how to use the app? How important would tech support be to troubleshoot with you if there are problems?
Perceived behavioral control	As described, how well do you think you could use the app to send your dietary supplement list to your clinician? Can you think of other barriers to using the app?
Perceived threat	Some dietary supplements interact with medications and can cause harm. How concerned are you about the risks associated with dietary supplements, particularly those your healthcare provider does not know you are taking?
Perceived benefits	How important do you think it might be to share your dietary supplement list with your healthcare provider with the mHealth app?
Social influence	How do you think your healthcare provider will feel about your use of the dietary supplement app to share your supplement list?
Performance expectancy	What do you think the dietary supplement mHealth app could do for you? How might the app make your visit with your healthcare provider easier if at all?
Privacy concerns	How concerned are you about your privacy in using the app? How would you feel if your dietary supplement information was shared without your knowledge?
Autonomy concerns	How strongly do you feel about sharing health information with your healthcare provider? Are there health habits you would not want to share? How do you feel about sharing information about your dietary supplement use with your provider?
Attitudes	How do you feel about the proposed dietary supplement app? How might the app meet your needs better?
Behavioral intention	If the dietary supplement app were available today, how likely would you be to use it? Why or why not?

Table S2 Codebook from Data Analysis

Code Group	Code
**Demographics**	Age
	Culture
	DS use
	Education
	Gender
	Provider type
	Race/ethnicity
**Why people use DS**	Anxiety
	Beauty
	Beliefs about health and disease
	Cancer
	Correct nutrient deficiency
	Culture
	Depression
	Doctor-recommended
	Fatigue
	General
	Health promotion
	Memory loss
	Pain
	Side effects of conventional medicines
	Sleep trouble/disturbance/insomnia
	Substitute for behavior/lifestyle change
	Substitute for medication
	Supplement availability
	Supplementing diet
	Weight lifting/muscle building
	Weight loss
**Safety/Threat of DS**	Adverse effects and DS-Disease interaction
	Beliefs about doctors
	Concern about supplement quality and safety
	Drug/supplement interactions
	Uncertainty about effects
	Wariness of advertising
**Autonomy**	Desire for control of health
	Patient willing to discuss diet/lifestyle
	Patient willing to discuss DS use with provider
	Reluctance to discuss DS
**Performance Expectancy**	Barcode scanning
	Connect to EMR

	Drug-supplement interaction checker
	General
	Notes
	Perceived overall value
	Reliable DS info
	Reminders
**Effort expectancy**	Effort expectancy/ease of app use
**Tech Self-Efficacy**	General
	Use of barcode scanner
	Tech self-efficacy by age or SES
	Technical facilitators of app use
**Potential App Benefit**	Communicating DS with provider
	Doctors' lack of training regarding DS
	Doctors' reluctance to discuss DS
	DS documentation challenges
	DS/medication recommendations
	Impact on clinical visit
	Improved/holistic care
	Lack of knowledge regarding DS
	Perceived benefit
	Providers specialized in DS
**Potential App Harm**	Cell phone connection and storage
	Cumbersome process
	Notifications/reminders
	Privacy related to app use
	Tech access by age or SES
	Unintended consequences
**Social Influence**	Friends, family, Internet recommended DS
	Provider asks about DS
	Provider disapproves of DS
	Provider doesn't ask about DS
	Provider recommends DS
	Provider support for app
**Behavioral Intention**	Behavioral intention/likeliness of app use
